# Surgical Cases Treated at the Hospital of the Sisters of Charity

**Published:** 1891-03

**Authors:** J. Henry Dowd

**Affiliations:** House Surgeon


### SURGICAL CASES TREATED AT THE HOSPITAL OF THE SISTERS OF CHARITY.

(SERVICE OF DR. MYNTER.)

REPORTED BY

J. HENRY DOWD, M. D., House Surgeon.

AMONG a large number of important operations lately performed at the Sisters of Charity Hospital, by Dr. H. Mynter, Professor of Surgery at Niagara University, the following deserve particular mention :

CASE I. *Partial Excision of the Sternum for Melanosarcom.* Mary F., 20 years of age, born in Ireland, entered the Sisters' Hospital on November 14, 1890, with the following history : She is a domestic and has been obliged to do a great deal of sweeping, during which the handle of the broom rubbed against the breast-bone. She ascribes her complaint — a tumor on the breast-bone — to this cause. The swelling first appeared eleven months ago, and has been growing larger steadily, till now it is the size of half an orange, and extends from above the third to below the fifth rib. Four months ago, the glands in the right axilla commenced to swell, and there is now found here a conglomeration of glands as large as two fists, completely filling the whole axilla, but yet somewhat movable. Two months ago the glands in the left axilla commenced to swell and are now as large as a hen's egg. During the last few weeks the glands in both supra-clavicular regions have commenced to enlarge. The tumor over the sternum is immovable, presents a feeling of false fluctuation ; the skin is normal in color and not adherent. She has sharp shooting pains radiating from the tumor in different directions, has lately commenced to lose flesh, but is yet in pretty good health. There are no symptoms of any growth in the anterior mediastinum, such as hoarseness, displacement of heart, difficulty of breathing or interference with circulation.

November 15th, under ether-narcosis, Dr. Mynter performed the following operation : U-formed incision, convex downwards, from second to sixth rib, about three inches wide. The flap was dissected up and the periosteum loosened from the sternum above the tumor in healthy tissue in order to shell out the tumor from the bone, if possible. The tumor was surrounded by a strong fibrous capsule. On a line of the fourth ribs the tumor extended into the breast-bone, and the capsule was necessarily opened here and there was a discharge of a semi-fluid, black, grumous substance. The rest of the tumor was, therefore, removed from below upwards by a similar process. The sternum was found quite extensively diseased, presenting an irregular cavity as large as a section of a hickory-nut, filled with the same black, grumous substance. It was thoroughly scraped out with a sharp spoon. During this process, the posterior lamina of the sternum was perforated. The opening was enlarged with chisels and from this opening the posterior periosteum was loosened with a curved elevator and the sternum removed piecemeal with cutting pliers, from the third to the fifth rib, inclusively. About one-half inch of the third, fourth, and fifth right costal cartilages were removed, too, and the pericardium, which appeared healthy, exposed for about six square inches. The skin flap was thereafter brought into place, sutured, and a drainage-tube inserted from the lowest point into the cavity.

All enlarged glands were thereafter removed from both axillæ and supra-clavicular regions, drainage-tubes introduced, sutures inserted, and antiseptic dressings applied. The operation lasted about two hours. The further course was favorable. The temperature rose for a couple of days to 101 in the evening. She suffered for some days considerable pain when breathing, till she learned to use abdominal respiration. The drains were removed on the fifth day and on the tenth day the wounds were healed and the patient allowed to sit up. She left the hospital on the fourteenth day.

Dr. W. C. Krauss examined the tumor microscopically and declared it a melanosarcom. Only two similar cases are reported in the literature, one by Kœnig and another by Küster. Kœnig tried to extirpate the sternum *in toto*, during which proceeding both pleural cavities and the pericardium were opened. His patient, nevertheless, recovered. This accident could scarcely happen if the operation is performed as in this case, *i. e.*, by trephining the sternum and removing the bone from the opening made with cutting pliers. So far, two months after the operation, no relapse has occurred, and the patient is greatly improved in health.

CASE II. *Double Castration, for Tuberculous Orchitis.* J. H., 50 years of age, entered the Sisters' Hospital on September 23, 1890, with the following history: Nine months ago he noticed that his left testicle was swelling. He is a contractor and has to ride a great deal over the rough pavement. He ascribes the testicular enlargement to injury from riding. The left testicle continued to grow larger and he consulted a physician, who punctured it for a hydrocele and injected tincture iodine. Four months ago the right testicle commenced to enlarge. On admittance the left testicle was found twice the size of normal, both the testis proper and the epididymis being enlarged. Two sinuses are present, through which a thick pus can be squeezed out. The right side of the scrotum is much enlarged, too, but the testis can be felt almost normal in size, the enlargement being caused by a hydrocele. He complains of severe pains in the back and groins. The family history is good; he has no cough nor symptoms of cachexia, no swollen glands in the neighborhood. The vesiculæ seminales seem normal.

September 24th, under ether-narcosis, Dr. Mynter opened the tunica vaginalis testis on the left side, and found it transformed into a pus-cavity. After scraping away diseased tissue two sinuses were found leading into the gland. It was then decided to remove it, which was done, the cord, which appeared healthy, being ligated near the external ring. The hydrocele on the right side was thereafter tapped and carbolic acid injected.

Dr. W. C. Krauss, who examined the extirpated gland, reported it to be a tuberculous orchitis and epididymitis and full of tuberculous bacilli.

He left the hospital recovered on October 1st, but returned on October 25th with the right testis in the same condition, swollen, painful, and with abscesses forming in it. He requested its removal, too, stating that he was unable to work, had a large family (nine children) to support and could stand the pain no longer. The right testis was, therefore, removed in a similar manner and the patient left recovered on November 1st, the wound being almost healed.

He returned again on December 6th on account of a considerable swelling, as large as a hen's egg, in the inguinal canal on the right side and a sinus on the same side, through which a little pus could be squeezed out. Under narcosis an extensive operation was performed, the inguinal canal being opened along its whole length to the internal ring and the tumor, which was a relapse along the cord, carefully dissected out. The inguinal canal was thereafter closed with strong catgut sutures. He left the hospital recovered on December 22d and has been well since.

The case is of importance on account of the patient's age, tuberculous orchitis generally occurring in early manhood, and on account of the rapid development of the disease.

Case III. *Spina Bifida.* Alice H., three weeks old, admitted November 12, 1890, with her mother. The baby, who is healthy-looking and with no other deformities, has a tumor, the size of a hen's egg, over the lumbo-sacral articulation covered with normal but very thin and tense skin and, apparently, being a pure meningocele. As the tumor was enlarging rapidly, it was considered best not to delay the operation in spite of the extreme youth, her age being only three weeks. Dr. Mynter therefore injected, without previous tapping, ʒi. of Morton's fluid (Iodine, gr. x.; Iodide Potash, ʒi.; Glycerine, ℥i.;) into the tumor, and painted the seat of puncture with collodium, over which firm pressure was applied. Some restlessness and coryza noted for some days. The child left the hospital on November 15th.

December 25th it was brought to the hospital, and the tumor was found to have entirely disappeared, its place being occupied by a dense cicatricial tissue.

This method offers by far better prospects of cure than excision, the percentage of recoveries being about 50 per cent. Two months is considered the limit of age, but that it may be safely done much earlier is well shown by this case.

CASE IV. *Resection of Maxilla Inferior.* D. H., 58 years of age, was admitted October 29, 1890, with the following history: About four months ago, while working in his blacksmith-shop, the handle of his hammer recoiled and struck him on the lower jaw. Since that time the body of the jaw has gradually enlarged and is now occupied by an epithelioma almost the size of a man's fist, and involving besides the whole body of the jaw from angle to angle a large part of the lower lip, the sublingual and submaxillary glands and the under surface of the tongue. The tumor gives him a peculiar appearance, his face being very much lengthened. His breath is extremely offensive, and a very fetid discharge is continually secreted from the mouth. He complains of intolerable pains radiating along his cheeks to the temples. Extensive cancerous ulcerations are seen and felt beneath the tongue. He is very much emaciated and cachectic from inability to swallow and from loss of sleep; there is marked calcification of the radial arteries and a large arcus senilis over both eyes. Both on account of this and of his general weak condition, operation was discouraged and the patient was honestly told that he probably would not survive an operation. As he, nevertheless, insisted upon taking his chances and have an operation performed, Dr. Mynter operated in the following manner: After preliminary tracheotomy, and after having plugged the pharynx with a large sponge, he split the lower lip and chin to the hyoid bone, dissected the healthy tissues away and severed the lower jaw with a chain-saw near both angles. Thereafter, turning the severed jaw downwards, he removed with scissors the whole cancerous *mass*, including the lower surface of the tongue and both submaxillary glands. The skin-flaps were thereafter sutured, with the exception of an opening near the hyoid bone, through which a drainage-tube was introduced. The tracheal-tube was removed on the twelfth day, the patient then being able to breathe through the mouth. He was nourished for four weeks with the stomach-tube, but is now able to swallow pretty well, although the rest of the tongue, by cicatricial retraction, is firmly drawn down to the floor of the mouth. His long face has been changed into a round, oval one, so that his best friends would scarcely recognize him. He has gained greatly in weight and strength, is free from pain, and able to enjoy life with comparative comfort.

CASE V. *Scirrhus of Rectum, and Excision of Rectum.* J. M., 41 years of age, was admitted on October 5, 1889, with the following history: He has for two years noticed hemorrhage from the bowels with every passage, preceded by an itching and burning sensation in the rectum. Last Summer he noticed that the passages became more frequent and accompanied not only with blood, but also with mucus, and with a painful sensation in the rectum as if he did not empty it completely. He was treated for piles by several physicians, but without any improvement. On examination a hard, firm lump, as large as a dove's egg, was found in the left half of the rectal wall including the sphincter and anus. Under ether-narcosis Dr. Mynter extirpated a rectangular piece of the rectum and anus including the tumor. About one-half of the circumference of the rectum was removed. The wound healed kindly and the patient was discharged in three weeks.

December 2, 1890, the patient was readmitted, complaining of pain and uneasiness in the rectum. He has felt perfectly well for a whole year, but the symptoms lately having reappeared, he returns for examination. A hard lump, as large as a hen's egg, was felt in the unremoved right half of the rectal wall, extending upwards almost three inches, and so immovable or firmly attached that it seemed to spring from the pelvic wall. Under ether-narcosis the right ischio-rectal fossa was opened by a large incision between the anus and tuberositas ischii. With much difficulty the tumor was loosened from the prostate and dissected loose from the surrounding tissues. It was not attached to the pelvic wall, but extended almost four inches upwards, and was removed with a good deal of difficulty and under profuse hemorrhage, more than thirty arteries being ligated in the wound, among which was the arteria pudenda communis. The vesico-rectal fossa was opened, and three or four nuckles of the small intestines prolapsed through the wound. The prolapsed intestines were replaced, three or four sutures taken in the cut end of the rectum and the peritoneum and two large drainage-tubes introduced into the vesico-rectal fossa. A very large tube, surrounded with iodoform gauze, was introduced into the rectum, and the whole wound firmly plugged with iodoform gauze around this tube. He vomited considerably for twenty-four hours and complained of pain in the abdomen, which was relieved by hypodermic injections of morphia; but the temperature did not rise above 101°, and was normal from the fourth day. The two smaller drainage-tubes were removed after forty-eight hours, the large one first on the tenth day, when firm adhesions were supposed to have taken place. His bowels were then opened by castor oil, and the use of daily hip-baths commenced. The large cavity filled up rapidly, and the cut end of the rectum was drawn down by the cicatricial retraction. He was out of bed in three weeks, and has since continually gained in strength. There is, of course, no control over the bowels, even the third sphincter having been removed. In order to prevent too much stenosis, a large rectal bougie is introduced daily.

[In a similar case I should think preliminary resection of a part of the sacrum, after Kraske's method, advisable. It gives a great deal more space and makes the operation easier, when the disease extends up to or above the vesico-rectal fold. Another point learned by this case is the necessity for local examination in all cases with symptoms of rectal disease. The diagnosis of piles is too often made without a local examination and cases of cancer overlooked, which might have been easily removed in the start and with a fair prospect of permanent cure.—H. M.]

CASE VI. *Double Osteotomy, on account of Bow-legs.* S. O., 25 years of age, born in England, was admitted on June 23, 1890, with the following history: Up to four years of age his legs were normal; but since then there has been a gradual bowing out of the knees and upper half of the tibia till twelve years of age, when the deformity ceased to increase. By examination a very peculiar deformity is seen. When his feet are placed together, both legs seem to form an arch, convex outwards, from the hips downwards, the inner measure between the knees being sixteen inches. If the knees are brought together, the feet and lower ends of the tibia cross each other, and the feet are over a foot apart crosswise. The curve is almost in the tibia and fibula alone, the femora being almost straight. A compensating curve, convex inwards, is seen in the lower third of the tibia, and as a further compensation both feet are in extreme *per valgus* position.

Under ether-narcosis, subcutaneous osteotomy, with Adam's saw, was performed on both tibia at the lowest end of the upper curve, the bones being sawn three-fourths through, the rest and the fibulæ then being fractured by direct force. The legs were thereafter straightened and put in plaster-of-Paris, an antiseptic bandage having first been applied. The feet were forced into a *per varus* position. August 26th the patient was discharged with good union and greatly improved in appearance, the inner measure between the knees now being only four inches.

---

THE *Albany Medical Annals* will, in the future, be conducted by the Albany Medical College Alumni Association, Dr. W. G. McDonald acting as editor. It will continue, as heretofore, the representative of the Alumni of Albany Medical College.—*St. Louis Medical and Surgical Journal.*

